# Evidence that Vitamin D Supplementation Could Reduce Risk of Influenza and COVID-19 Infections and Deaths

**DOI:** 10.3390/nu12040988

**Published:** 2020-04-02

**Authors:** William B. Grant, Henry Lahore, Sharon L. McDonnell, Carole A. Baggerly, Christine B. French, Jennifer L. Aliano, Harjit P. Bhattoa

**Affiliations:** 1 Sunlight, Nutrition, and Health Research Center, P.O. Box 641603, San Francisco, CA 94164-1603, USA; 2 2289 Highland Loop, Port Townsend, WA 98368, USA; hlahore@vitamindwiki.com; 3 GrassrootsHealth, Encinitas, CA 92024, USA; Sharon@grassrootshealth.org (S.L.M.); carole@grassrootshealth.org (C.A.B.); Christine@grassrootshealth.org (C.B.F.); jen@grassrootshealth.org (J.L.A.); 4 Department of Laboratory Medicine, Faculty of Medicine, University of Debrecen, Nagyerdei Blvd 98, H-4032 Debrecen, Hungary; harjit@med.unideb.hu

**Keywords:** acute respiratory distress syndrome (ARDS), ascorbic acid, cathelicidin, coronavirus, COVID-19, cytokine storm, influenza, observational, pneumonia, prevention, respiratory tract infection, solar radiation, treatment, UVB, vitamin C, vitamin D

## Abstract

The world is in the grip of the COVID-19 pandemic. Public health measures that can reduce the risk of infection and death in addition to quarantines are desperately needed. This article reviews the roles of vitamin D in reducing the risk of respiratory tract infections, knowledge about the epidemiology of influenza and COVID-19, and how vitamin D supplementation might be a useful measure to reduce risk. Through several mechanisms, vitamin D can reduce risk of infections. Those mechanisms include inducing cathelicidins and defensins that can lower viral replication rates and reducing concentrations of pro-inflammatory cytokines that produce the inflammation that injures the lining of the lungs, leading to pneumonia, as well as increasing concentrations of anti-inflammatory cytokines. Several observational studies and clinical trials reported that vitamin D supplementation reduced the risk of influenza, whereas others did not. Evidence supporting the role of vitamin D in reducing risk of COVID-19 includes that the outbreak occurred in winter, a time when 25-hydroxyvitamin D (25(OH)D) concentrations are lowest; that the number of cases in the Southern Hemisphere near the end of summer are low; that vitamin D deficiency has been found to contribute to acute respiratory distress syndrome; and that case-fatality rates increase with age and with chronic disease comorbidity, both of which are associated with lower 25(OH)D concentration. To reduce the risk of infection, it is recommended that people at risk of influenza and/or COVID-19 consider taking 10,000 IU/d of vitamin D_3_ for a few weeks to rapidly raise 25(OH)D concentrations, followed by 5000 IU/d. The goal should be to raise 25(OH)D concentrations above 40–60 ng/mL (100–150 nmol/L). For treatment of people who become infected with COVID-19, higher vitamin D_3_ doses might be useful. Randomized controlled trials and large population studies should be conducted to evaluate these recommendations.

## 1. Introduction

The world is now experiencing its third major epidemic of coronavirus (CoV) infections. A new CoV infection epidemic began in Wuhan, Hubei, China, in late 2019, originally called 2019-nCoV [1] and renamed COVID-19 by the World Health Organization on February 11, 2020. Previous CoV epidemics include severe acute respiratory syndrome (SARS)-CoV, which started in China in 2002 [2], and the ongoing Middle East respiratory syndrome (MERS)-CoV in the Middle East, first reported in 2012 [3]. Those epidemics all began with animal-to-human infection. The direct cause of death is generally due to ensuing severe atypical pneumonia [4,5]. 

Seasonal influenza has a high health burden. According to one recent estimate, 389,000 (uncertainty range 294,000–518,000) respiratory deaths were associated with influenza during the period 2002–2011 [6]. According to the U.S. Center for Disease Control and Prevention, during the period 2010–2019, annual symptomatic illness affected between 9 and 45 million people, resulting in between 4 and 21 million medical visits, 140,000–810,000 hospitalizations, and 23,000–61,000 deaths (https://www.cdc.gov/flu/about/burden/).

This review is a narrative one. Searches were made in PubMed.gov and scholar.google.com for publications regarding influenza, CoVs, COVID-19, and pneumonia with respect to epidemiology, innate and adaptive immune response, vitamin D, 25-hydroxyvitamin D (25(OH)D), and parathyroid hormone.

## 2. Vitamin D and Mechanisms to Reduce Microbial Infections

The general metabolism and actions of vitamin D are well known [7]. Vitamin D_3_ is produced in the skin through the action of UVB radiation reaching 7-dehydrocholesterol in the skin, followed by a thermal reaction. That vitamin D_3_ or oral vitamin D is converted to 25(OH)D in the liver and then to the hormonal metabolite, 1,25(OH)_2_D (calcitriol), in the kidneys or other organs as needed. Most of vitamin D’s effect arises from calcitriol entering the nuclear vitamin D receptor, a DNA binding protein that interacts directly with regulatory sequences near target genes and that recruits chromatin active complexes that participate genetically and epigenetically in modifying transcriptional output [8]. A well-known function of calcitriol is to help regulate serum calcium concentrations, which it does in a feedback loop with parathyroid hormone (PTH), which itself has many important functions in the body [7]. 

Several reviews consider the ways in which vitamin D reduces the risk of viral infections [9,10,11,12,13,14,15,16,17].

Vitamin D has many mechanisms by which it reduces the risk of microbial infection and death. A recent review regarding the role of vitamin D in reducing the risk of the common cold grouped those mechanisms into three categories: physical barrier, cellular natural immunity, and adaptive immunity [16]. Vitamin D helps maintain tight junctions, gap junctions, and adherens junctions (e.g., by E-cadherin) [18]. Several articles discussed how viruses disturb junction integrity, increasing infection by the virus and other microorganisms [19,20,21].

Vitamin D enhances cellular innate immunity partly through the induction of antimicrobial peptides, including human cathelicidin, LL-37, by 1,25-dihdroxyvitamin D [22,23], and defensins [24]. Cathelicidins exhibit direct antimicrobial activities against a spectrum of microbes, including Gram-positive and Gram-negative bacteria, enveloped and nonenveloped viruses, and fungi [25]. Those host-derived peptides kill the invading pathogens by perturbing their cell membranes and can neutralize the biological activities of endotoxins [26]. They have many more important functions, as described therein. In a mouse model, LL-37 reduced influenza A virus replication [27]. In another laboratory study, 1,25(OH)_2_D reduced the replication of rotavirus both in vitro and in vivo by another process [28]. A clinical trial reported that supplementation with 4000 IU/d of vitamin D decreased dengue virus infection [29].

Vitamin D also enhances cellular immunity, in part by reducing the cytokine storm induced by the innate immune system. The innate immune system generates both pro-inflammatory and anti-inflammatory cytokines in response to viral and bacterial infections, as observed in COVID-19 patients [30]. Vitamin D can reduce the production of pro-inflammatory Th1 cytokines, such as tumor necrosis factor α and interferon γ [31]. Administering vitamin D reduces the expression of pro-inflammatory cytokines and increases the expression of anti-inflammatory cytokines by macrophages ([17] and references therein).

Vitamin D is a modulator of adaptive immunity [16,32]; 1,25(OH)_2_D_3_ suppresses responses mediated by the T helper cell type 1 (Th1), by primarily repressing production of inflammatory cytokines IL-2 and interferon gamma (INFγ) [33]. Additionally, 1,25(OH)_2_D_3_ promotes cytokine production by the T helper type 2 (Th2) cells, which helps enhance the indirect suppression of Th1 cells by complementing this with actions mediated by a multitude of cell types [34]. Furthermore, 1,25(OH)_2_D_3_ promotes induction of the T regulatory cells, thereby inhibiting inflammatory processes [35].

Serum 25(OH)D concentrations tend to decrease with age [36], which may be important for COVID-19 because case-fatality rates (CFRs) increase with age [37]. Reasons include less time spent in the sun and reduced production of vitamin D as a result of lower levels of 7-dehydrocholesterol in the skin [38]. In addition, some pharmaceutical drugs reduce serum 25(OH)D concentrations by activating the pregnane-X receptor [39]. Such drugs include antiepileptics, antineoplastics, antibiotics, anti-inflammatory agents, antihypertensives, antiretrovirals, endocrine drugs, and some herbal medicines. Pharmaceutical drug use typically increases with age.

Vitamin D supplementation also enhances the expression of genes related to antioxidation (glutathione reductase and glutamate–cysteine ligase modifier subunit) [40]. The increased glutathione production spares the use of ascorbic acid (vitamin C), which has antimicrobial activities [41,42], and has been proposed to prevent and treat COVID-19 [43]. Moreover, a former director of the Center for Disease Control and Prevention, Dr. Tom Frieden, proposed using vitamin D to combat the COVID-19 pandemic on 23 March 2020 (https://www.foxnews.com/opinion/former-cdc-chief-tom-frieden-coronavirus-risk-may-be-reduced-with-vitamin-d).

## 3. Discussion

### 3.1. Seasonal Influenza

Influenza virus affects the respiratory tract by direct viral infection or by damage to the immune system response. The proximate cause of death is usually from the ensuing pneumonia. Patients who develop pneumonia are more likely to be < 5 years old, > 65 years old, white, and nursing home residents, to have chronic lung or heart disease and a history of smoking, and to be immunocompromised [44].

Seasonal influenza infections generally peak in winter [45]. Cannell et al. hypothesized that the winter peak was due in part to the conjunction with the season when solar UVB doses, and thus 25(OH)D concentrations, are lowest in most mid- and high-latitude countries [46], extended in [47]. Mean serum 25(OH)D concentrations in north and central regions of the United States are near 21 ng/mL in winter and 28 ng/mL in summer, whereas in the south region, they are near 24 ng/mL in winter and 28 ng/mL in summer [48]. In addition, the winter peak of influenza also coincides with weather conditions of low temperature and relative humidity that allow the influenza virus to survive longer outside the body than under warmer conditions [49,50,51].

Ecological studies suggest that raising 25(OH)D concentrations through vitamin D supplementation in winter would reduce the risk of developing influenza. Table 1 presents results from randomized controlled trials (RCTs) investigating how vitamin D supplementation affects risk of influenza. The RCTs included confirmed that the respiratory tract infection was indeed derived from influenza. Only two RCTs reported beneficial effects: one among schoolchildren in Japan [52], the other among infants in China [53]. An RCT in Japan that reported no beneficial effect did not measure baseline 25(OH)D concentration [54] and included many participants who had been vaccinated against influenza (M. Urashima; private communication). The two most recent RCTs included participants with above average mean baseline 25(OH)D concentrations [55,56]. A comprehensive review of the role of vitamin D and influenza was published in 2018 [15]. It concluded that the evidence of vitamin D’s effects on the immune system suggest that vitamin D should reduce the risk of influenza, but that more studies are required to evaluate that possibility. Large population studies would also be useful, in which vitamin D supplementation is also related to changes in serum 25(OH)D concentration.

An observational study conducted in Connecticut on 198 healthy adults in the fall and winter of 2009–2010 examined the relationship between serum 25(OH)D concentration and incidence of acute RTIs (ARTIs) [57]. Only 17% of people who maintained 25(OH)D >38 ng/mL throughout the study developed ARTIs, whereas 45% of those with 25(OH)D < 38 ng/mL did. Concentrations of 38 ng/mL or more were associated with a significant (*p* < 0.0001) twofold reduction in risk of developing ARTIs and with a marked reduction in the percentage of days ill. Eight influenza-like illnesses (ILIs) occurred, seven of which were the 2009 H1N1 influenza.

### 3.2. Clinical and Epidemiological Findings Regarding COVID-19

The first step in developing a hypothesis is to outline the epidemiological and clinical findings regarding the disease of interest and their relationship with 25(OH)D concentrations. From the recent journal literature, it is known that COVID-19 infection is associated with the increased production of pro-inflammatory cytokines [58], C-reactive protein [30], increased risk of pneumonia [58], sepsis [59], acute respiratory distress syndrome [59], and heart failure [59]. CFRs in China were 6%–10% for those with cardiovascular disease, chronic respiratory tract disease, diabetes, and hypertension [37]. Two regions hard hit by COVID-19 are regions of high air pollution in China [60] and northern Italy [61]. 

The possible roles of vitamin D for the clinical and epidemiological characteristics of diseases associated with the increased risk of COVID-19 CFR are given in Table 2. Most of the beneficial effects of vitamin D given in Table 2 are from observational studies of disease incidence or prevalence with respect to serum 25(OH)D concentrations. RCTs comparing outcomes for participants treated or given a placebo are preferred to establish causality related to health outcomes. However, most vitamin D RCTs have not reported that vitamin D supplementation reduced the risk of disease [62,63]. Reasons for the lack of agreement between observational studies and RCTs seems to be due to several factors, including enrolling participants with relatively high 25(OH)D concentrations and using low vitamin D doses and not measuring baseline and achieved 25(OH)D concentrations. Previous studies proposed that RCTs of nutrients such as vitamin D be based on nutrient status, such as 25(OH)D concentration, seeking to enroll participants with low values, supplementing them with enough agent to raise the concentration to values associated with good health, and measuring achieved concentrations as well as cofactors such as vitamin C, omega-3 fatty acids, and magnesium [64,65],. Two recently completed RCTs reported significantly reduced incidence in the secondary analyses for cancer [66] and diabetes mellitus [67].

Table 3 lists some findings for vitamin D supplementation in reducing the clinical effects of COVID-19 infection found from treating other diseases.

A possible reason for the monotonic increase in CFR with increasing age could be that the presence of chronic diseases increases with age. For example, the global prevalence of diabetes mellitus increases from about 1% below the age of 20 years, to ~10% at 45 years and to 19% at 65 years, decreasing to 14% by 95 years [94]. Invasive lung cancer incidence rates for females in the United States in 2015 increased from 1.1/100,000 for those aged 30–34 years, to 51.0/100,000 for those aged 50–54 years, 204.1/100,000 for those aged 65–79 years, and 347.3 for those aged 75–79 years [95]. Several studies report that people with chronic diseases have lower 25(OH)D concentrations than healthy people. A study in Italy reported that male chronic obstructive pulmonary disease patients had mean 25(OH)D concentrations of 16 (95% CI, 13–18) ng/mL, whereas female patients had concentrations of 13 (95% CI, 11–15) ng/ml [96]. A study in South Korea reported that community-acquired pneumonia (CAP) patients had a mean 25(OH)D concentration at admission of 14 ± 8 ng/mL [97]. A study in Iran reported that hypertensive patients had lower 25(OH)D concentrations than control subjects: males, 13 ± 11 vs. 21 ± 11 ng/mL; females, 13 ± 10 vs. 20 ± 11 ng/mL [98].

Another factor that affects immune response with age is reduced 1,25-dihydroxyvitamin D (1,25(OH)_2_D, or calcitriol), the active vitamin D metabolite, with increased age. Parathyroid hormone (PTH) concentration increases with age. A U.S. study was based on 312,962 paired serum PTH and 25(OH)D concentration measurements from July 2010 to June 2011. For participants with 20-ng/mL 25(OH)D concentration, PTH increased from 27 pg/mL for those <20 years to 54 pg/mL for those >60 years [99]. Serum calcitriol concentrations are inversely related to PTH concentrations. In a study conducted in Norway on patients with a mean age of 50 (SD, 21) years, calcitriol decreased from 140 pmol/L for those aged 20–39 years to 98 pmol/L for those >80 years despite an increase in serum 25(OH)D from 24 ng/mL for those 20–39 years to 27 ng/mL for those >80 years [100].

The seasonality of many viral infections is associated with low 25(OH)D concentrations, as a result of low UVB doses owing to the winter in temperate climates and the rainy season in tropical climates—such as respiratory syncytial virus (RSV) infection [101,102],. This is the case for influenza [45,46], and SARS-CoV [103]. However, MERS showed a peak in the April–June quarter [104], probably affected by both Hajj pilgrims gathering and the fact that 25(OH)D concentrations show little seasonal variation in the Middle East [105]. In the tropics, seasonality is related more to rainy periods (low UVB doses), for example, for influenza [106].

Considerable indirect evidence is inferred from effects found for other enveloped viruses. Table 4 presents the findings from various studies.

One way that CoVs injure the lung epithelial cells and facilitate pneumonia is through increased production of Th1-type cytokines as part of the innate immune response to viral infections, giving rise to the cytokine storm. A laboratory cell study reported that interferon γ is responsible for acute lung injury during the late phase of the SARS-CoV pathology [117].

Pro-inflammatory cytokine storms from CoV infections have resulted in the most severe cases for SARS-CoV [118] and MERS-CoV [119]. However, COVID-19 infection also initiated increased secretion of the Th2 cytokines (e.g., interleukins 4 and 10) that suppress inflammation, which differs from SARS-CoV infection [30].

### 3.3. Pneumonia 

An example of the role of vitamin D in reducing the risk of death from pandemic respiratory tract infections is found in a study of CFRs resulting from the 1918–1919 influenza pandemic in the United States [120]. The U.S. Public Health Service conducted door-to-door surveys of 12 communities from New Haven, Connecticut, to San Francisco, California, to ascertain incidence and CFRs. The canvasses were made as soon as possible after the autumn 1918 wave of the epidemic subsided in each locality. A total of 146,203 people, 42,920 cases, and 730 deaths were found. As shown in their Table 25, fatality rates averaged 1.70 per 100 influenza cases but averaged 25.5 per 100 cases of pneumonia. The percentage of influenza complicated by pneumonia was 6.8%. The pneumonia CFR (excluding Charles County, MD, because of inconsistencies in recording cause of death) was 28.8 per 100 for whites and 39.8 per 100 for “coloreds”. As shown in Table 23, “coloreds” in the southeastern states had between a 27% and 80% higher rate of pneumonia compared to whites. As discussed in an ecological study using those CFR data, communities in the southwest had lower CFR than those in the northeast because of higher summertime and wintertime solar UVB doses [121]. Previous work suggested that higher UVB doses were associated with higher 25(OH)D concentrations, leading to reductions in the cytokine storm and the killing of bacteria and viruses that participate in pneumonia. African Americans had much higher mortality rates than white Americans for the period 1900–1948 [122]. The reasons CFRs were higher for “coloreds” than whites may include that they have higher rates of chronic diseases, are more likely to live in regions impacted by air pollution, and that with darker skin pigmentation, blacks have lower 25(OH)D concentrations. A clinical trial involving postmenopausal women living on Long Island, NY with mean baseline 25(OH)D concentration 19 ± 8 ng/mL found that supplementation with 2000 IU/d resulted in significantly fewer upper respiratory tract infections, including influenza, than a placebo or supplementation with 800 IU/d [123]. See, also, references in [11]. An analysis of serum 25(OH)D concentrations by race for 2001–2004 indicated mean 25(OH)D concentrations for people over 40 years: non-Hispanic whites, ~25–26 ng/mL; non-Hispanic blacks, 14–17 ng/mL; Mexican–Americans, 18–22 ng/mL [124]. A reason proposed for the higher mortality rates in some communities during the 1918–1919 influenza pandemic was that they were near to coal-fired electricity generating plants [125]. Recent studies have confirmed that air pollution, from combustion sources, increases the risk of influenza [126,127]. The highest concentration of these plants is in the northeast, where solar UVB doses are lowest.

A high-dose (250,000 or 500,000 IU) vitamin D_3_ trial in ventilated intensive care unit patients in Georgia with mean a baseline 25(OH)D concentration of 20–22 ng/mL reported that hospital length of stay was reduced from 36 (SD, 19) days in the control group to 25 (SD, 14) days in the 250,000-IU group [25(OH)D = 45 ± 20 ng/mL] and 18 (SD, 11) days in the 500,000-IU group [25(OH)D = 55 ± 14 ng/mL]; *p* = 0.03 [93]. In a follow-on pilot trial involving 30 mechanically ventilated critically ill patients, 500,000 IU of vitamin D_3_ supplementation significantly increased hemoglobin concentrations and lowered hepcidin concentrations, improving iron metabolism and the blood’s ability to transport oxygen [128].

## 4. Recommendations

### 4.1. Hospital-Acquired Infections

Hospitals are a source of RTIs for both patients and medical personnel. For example, during the SARS-CoV epidemic, a woman returned to Toronto from Hong Kong with SARS-CoV in 2003 and went to a hospital. The disease was transmitted to other people, leading to an outbreak among 257 people in several Greater Toronto Area hospitals [129]. During the 2014–2015 influenza season, 36% of health care workers in a German hospital developed influenza infection [130].

Working in a hospital dealing with COVID-19 patients is associated with increased risk of COVID-19 infection. For example, 40 of 138 hospitalized COVID-19 patients in Wuhan in the Zhongnan Hospital from 1 to 28 January were medical staff, and 17 more were infected while in the hospital [58]. It was announced on February 14, 2020, that more than 1700 Chinese health workers were infected by COVID-19 and six had died (https://www.huffpost.com/entry/chinese-health-workers-infected-by virus_n_5e46a0fec5b64d860fc97c1b).

Vitamin D supplementation to raise serum 25(OH)D concentrations can help reduce hospital-associated infections [131]. Concentrations of at least 40–50 ng/mL (100–125 nmol/L) are indicated on the basis of observational studies [132,133]. During the COVID-19 epidemic, all people in the hospital, including patients and staff, should take vitamin D supplements to raise 25(OH)D concentrations as an important step in preventing infection and spread. Trials on that hypothesis would be worth conducting.

### 4.2. Proposed Actions

The data reviewed here supports the role of higher 25(OH)D concentrations in reducing risk of infection and death from ARTIs, including those from influenza, CoV, and pneumonia. The peak season for ARTIs is generally when 25(OH)D concentrations are lowest. Thus, vitamin D_3_ supplementation should be started or increased several months before winter to raise 25(OH)D concentrations to the range necessary to prevent ARTIs. Studies reviewed here generally reported that 25(OH)D concentrations of 20–30 ng/mL reduced the risk of ARTIs [134]. One reason for that result may be that the studies included few participants with higher 25(OH)D concentrations. However, one observational study reported that 38 ng/mL was the appropriate concentration to reduce the risk of CAP [57]. Although the degree of protection generally increases as 25(OH)D concentration increases, the optimal range appears to be in the range of 40–60 ng/mL (100–150 nmol/l). To achieve those levels, approximately half the population could take at least 2000–5000 IU/d of vitamin D_3_ [135]. Various loading doses have been studied for achieving a 25(OH)D concentration of 30 ng/mL. For example, one study used a weekly or fortnightly dose totaling 100,000–200,000 IU over 8 weeks (1800 or 3600 IU/d) [136]. However, to achieve 40–60 ng/mL would take higher loading doses. A trial involving Canadian breast cancer patients with bone metastases treated with bisphosphonates but without comorbid conditions reported that doses of 10,000 IU/d of vitamin D_3_ over a four-month period showed no adverse effects, but did unmask two cases of primary hyperparathyroidism [137]. A study involving 33 participants, including seven taking 4000 IU/d of vitamin D_3_ and six who took 10,000 IU/d of vitamin D_3_ for 8 weeks, reported that 25(OH)D concentrations increased from 20 ± 6 to 39 ± 9 for 4000 IU/d and from 19 ± 4 to 67 ± 3 for 10,000 IU/d and improved gut microbiota with no adverse effects [138]. Thus, from the literature, it is reasonable to suggest taking 10,000 IU/d for a month, which is effective in rapidly increasing circulating levels of 25(OH)D into the preferred range of 40–60 ng/mL. To maintain that level after that first month, the dose can be decreased to 5000 IU/d [135,139,140]. When high doses of vitamin D are taken, calcium supplementation should not be high to reduce risk of hypercalcemia.

A recent review suggested using vitamin D loading doses of 200,000–300,000 IU in 50,000-IU capsules to reduce the risk and severity of COVID-19 [43].

The efficacy and safety of high-dose vitamin D supplementation has been demonstrated in a psychiatric hospital in Cincinnati, Ohio [141]. The age range was from 18 to 90 years. Half of the patients were black, and nearly half were white. All patients entering since 2011 were offered supplementation of 5000 or 10,000 IU/d vitamin D_3_. For 36 patients who received 5000 IU/d for 12 months or longer, mean serum 25(OH)D concentration rose from 24 to 68 ng/mL, whereas for the 78 patients who received 10,000 IU/d, mean concentrations increased from 25 to 96 ng/mL. No cases of vitamin D–induced hypercalcemia were reported. This article includes a brief review of other high-dose vitamin D studies, including the fact that vitamin D doses of 60,000–600,000 IU/d were found to treat and control such diseases as asthma, rheumatoid arthritis, rickets, and tuberculosis in the 1930s and 1940s. Those doses are much higher than the 10,000–25,000 IU/d of vitamin D_3_ that can be made from solar UVB exposure [142]. However, after reports of hypercalcemia associated with use of supra-physiological doses of vitamin D surfaced, e.g., [143], high-dose vitamin D supplementation fell out of favor.

A recent article on a high-dose vitamin D supplementation trial in New Zealand involving 5110 participants reported that, over a median of 3.3 years, monthly supplementation with 100,000 IU of vitamin D_3_ did not affect the incidence rate of kidney stone events or hypercalcemia [144].

Unfortunately, most countries do not have guidelines supporting vitamin D supplementation doses and desirable serum 25(OH)D concentrations that would deal with wintertime RTIs. Guidelines for many countries consider 20 ng/mL (50 nmol/L) adequate. According to the statement from the European Society for Clinical and Economic Aspects of Osteoporosis, Osteoarthritis, and Musculoskeletal Diseases, “attainment of serum 25-hydroxyvitamin D levels well above the threshold desired for bone health cannot be recommended based on current evidence, since safety has yet to be confirmed” [145]. This statement, published in 2017, is no longer correct since a number of vitamin D supplementation studies have reported that long-term vitamin D supplementation has health benefits without adverse health effects, e.g., 2000 IU/d for cancer risk reduction [66,146] and 4000 IU/d for reduced progression from prediabetes to diabetes [67].

A recent review on the status of vitamin D deficiency worldwide stated that because of inadequate evidence from clinical trials, “a 25(OH)D level of >50 nmol/L or 20 ng/mL is, therefore, the primary treatment goal, although some data suggest a benefit for a higher threshold” [147]. A companion article in the same issue of the journal stated, “although 20 ng/mL seems adequate to reduce risk of skeletal problems and ARTIs, concentrations above 30 ng/mL have been associated with reduced risk of cancer, type 2 diabetes mellitus, and adverse pregnancy and birth outcomes” [148]. However, on the basis of the findings in several studies discussed here, as well as recommendations for breast and colorectal cancer prevention [149], the desirable concentration should be at least 40–60 ng/mL.

The U.S. Institute of Medicine issued vitamin D and calcium guidelines in 2011 [150]. The institute recommended vitamin D supplementation of 600 IU/d for people younger than 70 years, 800 IU/d for those older than 70 years, and a serum 25(OH)D concentration of 20 ng/mL (50 nmol/L) or higher. That recommendation was based on the effects of vitamin D for bone health. The institute recognized that no studies had reported adverse effects of supplementation with less than 10,000 IU/d of vitamin D, but set the upper intake level at 4000 IU/d, partly out of concerns stemming from observational studies that found U-shaped 25(OH)D concentration–health outcome relationships. However, later investigation determined that most reports of J- or U-shaped relationships were from observational studies that did not measure serum 25(OH)D concentrations and that the likely reason for those relationships was a result of enrolling some participants who had started taking vitamin D supplements shortly before enrolling [151].

Moreover, in 2011, the Endocrine Society recommended supplementation of 1000–4000 IU/d of vitamin D and a serum 25(OH)D concentration of 30 ng/mL or higher [152]. Those guidelines were for patients. It appears that anyone with chronic disease should be considered in that category. The U.S. Institute of Medicine noted that no adverse effects of vitamin D supplementation had been reported for daily doses <10,000 IU/d [150].

Measuring serum 25(OH)D concentration would be useful to determine baseline and achieved 25(OH)D concentrations. A recent article recommended testing for groups of people who were likely to have low concentrations and could benefit from higher concentrations, such as pregnant women, the obese, people with chronic diseases, and the elderly [148]. Part of the rationale for testing was to increase awareness of actual 25(OH)D concentrations and the benefits of higher concentrations. In addition, increases in 25(OH)D concentration with respect to vitamin D supplementation depend on various personal factors, including genetics, digestive system health, weight, and baseline 25(OH)D concentration. For about half the population, taking 5000 IU/d of vitamin D_3_ or 30,000–35,000 IU/wk would raise 25(OH)D concentration to 40 ng/mL. Taking 6235–7248 IU/d as proposed to ensure that 97.5% of the population has concentrations >20 ng/mL [153] would not exceed the 10,000-IU/d threshold.

Vitamin D supplementation is required for many individuals to reach 25(OH)D concentrations above 30 ng/mL, especially in winter [154]. However, vitamin D fortification of basic foods such as dairy and flour products [83,155] can raise serum 25(OH)D concentrations of those members of various populations with the lowest concentrations by a few ng/mL. Doing so can result in reduced risk of ARTIs for individuals with extreme vitamin D deficiency [134,156]. However, for greater benefits, daily or weekly vitamin D supplementation is recommended [134], as is the annual determination of serum 25(OH)D concentration for those with health risks [148].

Magnesium supplementation is recommended when taking vitamin D supplements. Magnesium helps activate vitamin D, which in turn helps regulate calcium and phosphate homeostasis to influence the growth and maintenance of bones. All the enzymes that metabolize vitamin D seem to require magnesium, which acts as a cofactor in the enzymatic reactions in the liver and kidneys [157]. The dose of magnesium should be in the range of 250–500 mg/d, along with twice that dose of calcium.

The hypothesis that vitamin D supplementation can reduce the risk of influenza and COVID-19 incidence and death should be investigated in trials to determine the appropriate doses, serum 25(OH)D concentrations, and the presence of any safety issues. The RCT on vitamin D supplementation for ventilated ICU patients conducted in Atlanta, Georgia, is a good model [93].

A recent review stated: “Although contradictory data exist, available evidence indicates that supplementation with multiple micronutrients with immune-supporting roles may modulate immune function and reduce the risk of infection. Micronutrients with the strongest evidence for immune support are vitamins C and D and zinc. Better design of human clinical studies addressing dosage and combinations of micronutrients in different populations are required to substantiate the benefits of micronutrient supplementation against infection.” [17].

## Figures and Tables

**Table 1:** Results of vitamin D randomized controlled trials (RCTs) on risk of influenza.

Country	Population	Baseline 25(OH)D (ng/mL)	Vitamin D Dose (IU/d)	Influenza Cases in Vitamin D, Placebo Arms	Outcome	Ref
Japan	Schoolchildren aged 6–15 yrs	N/A	0, 1200	Type A: 18/167; 31/167. If not taking vitamin D before enrollment: 8/140; 22/140. Type B: 39/167; 28/167	Type A: RR = 0.58 (95% CI, 0.34 to 0.99); if not taking vitamin D before enrollment, RR = 0.36 (95% CI, 0.17 to 0.79); no effect for Type B	[52]
Japan	High school students, including many vaccinated against influenza	N/A	0, 2000	20/148; 12/99	Type A, RR = 1.11 (95% CI, 0.57 to 2.18)	[54]
China	Infants, 3–12 mos	17	400, 1200		Diff. in influenza A viral load, high vs. low vitamin D on day 4 of illness: 1.3 ± 0.5 vs. 4.5 ± 1.4 × 10^6^ copies/mL	[53]
Japan	223 patients with IBD, mean age 45 yrs	23–24	0, 500	8/115; 6/108	RR = 1.25 (95% CI, 0.45 to 3.49)	[55]
Vietnam	Children aged 3–17 yrs	26	0, 14,000 /wk	50/650; 43/650	HR = 1.18 (95% CI, 0.79 to 1.78)	[56]

Note: 95% confidence interval (95% CI); day (d); hazard ratio (HR); inflammatory bowel disease (IBD); months (mos); not available (N/A); relative risk (RR); upper respiratory tract infection (URTI); week (wk); years (yrs).

**Table 2:** How vitamin D is related to the clinical and epidemiological findings for incidence and case-fatality rates.

Characteristics	Relation to 25(OH)D	Reference
Clinical		
Severe cases associated with pneumonia	Inverse correlation for CAP	[68,69]
Increased production of pro-inflammatory cytokines such as IL-6	Inverse correlation	[70,71]
Increased CRP	Inverse correlation	[72,73]
Increased risk of sepsis	Inverse correlation	[74,75]
Risk of ARDS	Inverse correlation	[76,77]
Risk of heart failure	Inverse correlation	[78,79]
Risk of diabetes mellitus	Inverse correlation	[67,80]
Epidemiological		
Began in December 2019 in China, spread mainly to northern midlatitude countries	Low 25(OH)D values in winter	[48,81]
Males have higher incidence and much higher CFRs than females	Smoking reduces 25(OH)D	[82]
CFR increases with age	Chronic disease rates increase with age; vitamin D plays a role in reducing risk of chronic diseases	[83]
Higher CFR for diabetics	Diabetics may have lower 25(OH)D	[84]
Higher CFR for diabetics	Lower 25(OH)D associated with increased risk of incidence	[85]
Higher CFR for hypertension	Lower 25(OH)D may be associated with increased risk of incidence	[86]
Higher CFR for cardiovascular disease	Lower 25(OH)D associated with increased risk of incidence and death	[87]
Higher CFR for chronic respiratory disease	For COPD patients, 25(OH)D inversely correlated with risk, severity, and exacerbation	[88]
Found at higher rates in regions with elevated air pollution	Air pollution associated with lower 25(OH)D concentrations	[89]

Note: 25-hydroxyvitamin D ((25(OH)D); acute respiratory distress syndrome (ARDS); community-acquired pneumonia (CAP); case-fatality rate (CFR); interleukin 6 (IL-6); chronic obstructive pulmonary disease (COPD); C-reactive protein (CRP); vitamin D deficiency (VDD).

**Table 3:** How vitamin D supplementation is related to the clinical and epidemiological findings for treatment.

Clinical Characteristics	Findings from Vitamin D Supplementation Trials	Reference
Treatment of CAP with vitamin D	Did not significantly result in complete resolution. Baseline 25(OH)D was 20 ng/ml. Achieved 25(OH)D in the treatment arm was 40 ng/mL.	[90]
Increased production of pro-inflammatory cytokines such as IL-6	Reduces concentration of IL-6	[11]
Increased CRP	Reduces CRP in diabetic patients	[91]
Increased risk of sepsis	No reduction in mortality rate found for adults with sepsis supplemented with vitamin D. Most trials included participants with 25(OH)D <20 ng/mL; vitamin D_3_ doses between 250 and 600 thousand IU.	[92]
Risk of ARDS	Vitamin D deficiency contributes to development of ARDS	[77,93]

Acute respiratory distress syndrome (ARDS); community-acquired pneumonia (CAP); case-fatality rate (CFR); interleukin 6 (IL-6); chronic obstructive pulmonary disease (COPD); C-reactive protein (CRP); vitamin D deficiency (VDD).

**Table 4:** Findings regarding the associations and effects of vitamin D on enveloped viral infections.

Virus	Vitamin D Effect	Reference
Dengue	Vitamin D mechanisms discussed	[107]
Dengue	Inverse association between 25(OH)D concentration and progression of disease state	[108]
Dengue	Vitamin D supplementation trial with 1000 and 4000 IU/d. 4000 IU/d resulted in higher resistance to DENV-2 infection. MDDCs from those supplemented with 4000 IU/d showed decreased mRNA expression of TLR3, 7, and 9; downregulation of IL-12/IL-8 production; and increased IL-10 secretion in response to DENV-2 infection	[29]
Hepatitis C	1,25-hydroxyvitamin-D3-24-hydroxylase, encoded by CYP24A1 gene, is a key enzyme that neutralizes 1,25(OH)_2_D. This study found that alleles of CYP24A1 had different effects on risk of chronic hepatitis C infection.	[109]
CHB	25(OH)D concentrations were lower in CHB patients than that of healthy controls and inversely correlated with HBV viral loads	[110]
KSHV	Found that cathelicidin significantly reduced KSVH by disrupting the viral envelope.	[111]
HIV-1	Review of 29 clinical studies of vitamin D supplementation showed there was a decrease in inflammation. In 3 of 7 studies, CD4+ T cell count increased, but effect on viral load was inconclusive since most patients were on cART.	[112]
H9N2 influenza	In a lung epithelial cell study, calcitriol treatment prior to and post infection with H9N2 influenza significantly decreased expression of the influenza M gene, IL-6, and IFN-β in A549 cells, but did not affect virus replication.	[113]
RSV	Demonstrated that the human cathelicidin LL-37 has effective antiviral activity against RSV in vitro and prevented virus-induced cell death in epithelial cultures,	[114]
RSV	Performed a laboratory study that identified the mechanism by which vitamin D reduced risk of RSV.	[28]
RSV	Found that the T-allele of the vitamin D receptor has a lower prevalence in African populations and runs parallel to the lower incidence of RSV-associated severe ALRI in African children, 1 year.	[115]
Rotaviral diarrhea	Found serum 25(OH)D <20 ng/mL associated with an odds ratio of 6.3 (95% CI, 3.6 to 10.9) for rotaviral diarrhea	[116]

Note: acute respiratory tract infection (ALRI); combination Antiretroviral Therapy (cART); chronic hepatitis B (CHB); dengue virus-2 (DENV-2). Human immunodeficiency virus 1 (HIV-1); Kaposi’s sarcoma-associated herpesvirus (KSHV); monocyte-derived dendritic cells (MDDCs); respiratory syncytial virus (RSV).
